# The Beneficial Effect of Self-Compassion on PTSD and Complex PTSD Symptoms among Israeli Female Veterans: The Role of Coping Strategies

**DOI:** 10.1192/j.eurpsy.2025.1970

**Published:** 2025-08-26

**Authors:** G. Zerach

**Affiliations:** 1 Psychology, Ariel University, Ariel, Israel

## Abstract

**Introduction:**

There is consistent evidence that increased self-compassion (SC) is associated with less posttraumatic stress disorder (PTSD) symptoms. However, knowledge about the contribution of SC to military-related posttraumatic sequelae among women combat veterans is sparse. Moreover, the underlying mechanism for the beneficial effect of SC remains to be determined.

**Objectives:**

The present study aims to examine the contribution of SC to PTSD and complex PTSD symptoms among female veterans as well as the mediating roles of coping strategies in these possible associations.

**Methods:**

In a cross-sectional study, a volunteer community sample of Israeli women combat veterans (n = 885) and non-combat veterans (n = 728) responded to online self-report questionnaires.

**Results:**

Combat veterans reported higher levels of PTSD symptoms but not complex PTSD symptoms, SC, or coping strategies, as compared to non-combat veterans. Moreover, among combat and noncombat veterans, SC was associated with lower levels of PTSD and complex PTSD symptoms beyond adverse childhood experiences and combat exposure. Notably, SC was inversely and indirectly associated with higher levels of PTSD and complex PTSD symptoms through maladaptive coping strategies for both combat and noncombat veterans.

**Conclusions:**

Reports of higher SC among female veterans are associated with less severe PTSD and complex PTSD symptoms. Moreover, maladaptive coping strategies might serve as mechanisms that link SC to military-related posttraumatic consequences.

**Disclosure of Interest:**

None Declared

